# Consensus classification of human leukocyte antigen class II proteins

**DOI:** 10.1007/s00251-012-0665-6

**Published:** 2012-11-16

**Authors:** Indrajit Saha, Giovanni Mazzocco, Dariusz Plewczynski

**Affiliations:** 1 Interdisciplinary Centre for Mathematical and Computational Modeling, University of Warsaw, 02-106 Warsaw, Poland; 2 Department of Computer Science and Engineering, Jadavpur University, Kolkata, 700032 India

**Keywords:** MHC, HLA class II, Peptide binding, T cell epitopes, Clustering, Machine learning

## Abstract

Class II human leukocyte antigens (HLA II) are proteins involved in the human immunological adaptive response by binding and exposing some pre-processed, non-self peptides in the extracellular domain in order to make them recognizable by the CD4+ T lymphocytes. However, the understanding of HLA–peptide binding interaction is a crucial step for designing a peptide-based vaccine because the high rate of polymorphisms in HLA class II molecules creates a big challenge, even though the HLA II proteins can be grouped into supertypes, where members of different class bind a similar pool of peptides. Hence, first we performed the supertype classification of 27 HLA II proteins using their binding affinities and structural-based linear motifs to create a stable group of supertypes. For this purpose, a well-known clustering method was used, and then, a consensus was built to find the stable groups and to show the functional and structural correlation of HLA II proteins. Thus, the overlap of the binding events was measured, confirming a large promiscuity within the HLA II–peptide interactions. Moreover, a very low rate of locus-specific binding events was observed for the HLA-DP genetic locus, suggesting a different binding selectivity of these proteins with respect to HLA-DR and HLA-DQ proteins. Secondly, a predictor based on a support vector machine (SVM) classifier was designed to recognize HLA II-binding peptides. The efficiency of prediction was estimated using precision, recall (sensitivity), specificity, accuracy, F-measure, and area under the ROC curve values of random subsampled dataset in comparison with other supervised classifiers. Also the leave-one-out cross-validation was performed to establish the efficiency of the predictor. The availability of HLA II–peptide interaction dataset, HLA II-binding motifs, high-quality amino acid indices, peptide dataset for SVM training, and MATLAB code of the predictor is available at http://sysbio.icm.edu.pl/HLA.

**Electronic supplementary material:**

The online version of this article (doi:10.1007/s00251-012-0665-6) contains supplementary material, which is available to authorized users.

## Introduction

Antigen presentation is the crucial process for eliciting an efficient immune response since T cells fail to recognize non-self antigens in the absence of the human leukocyte antigen (HLA)–peptide complex (Vivona et al. 2008). The T cell receptor is restricted to identifying antigenic peptides only when bound to find suitable HLA molecules. HLA binding antigens can be generated by the exogenous pathogen pathway, which is operated by specialized antigen-presenting cells (APC) to initiate and promote the development of lymphocyte activation. Exogenous antigens must be internalized by the APC, digested into small peptides, and bound to the peptide-binding groove of the HLA II molecules, in order to be recognized by antigen-specific CD4+ T cells (Vivona et al. 2008).

The HLA system is characterized by an extremely high level of polymorphism resulting in highly comprehensive antigen presentation. This is more relevant in the HLA class II, where different gene loci are coded in α (DRA, DQA, and DPA) and β (DRB, DQB, and DPB) chains. There is a fundamental difference in structural composition between HLA class I and HLA class II proteins, resulting in very different binding characteristics (Vivona et al. 2008). In the HLA class I, the binding groove is closed at both ends, allowing the binding of only nine amino acid long peptides using a unique binding frame (Vivona et al. 2008). The peptide-binding core of HLA class II molecules is open at both ends. Therefore, the size of peptides that can bind the groove typically ranges from 12 to 24 amino acid residues. Moreover, each peptide can bind different open binding grooves by using different binding registers. This wide variability largely complicates the binding predictions (Gowthaman and Agrewala 2008). Both chains of the HLA II molecule interact with the side chains of the peptide and determine binding affinity, but the majority of the polymorphic residues are located within the β chain. Moreover, each class II allele has different side chain contacts, which allow only peptides with certain amino acids to bind into particular key positions, called anchor positions. The peptide anchor position closest to the N-terminal accepts hydrophobic residues by including large aromatic amino acids that are essential for binding peptides with high affinity (Dai et al. 2010). These anchors are the amino acids mostly found in peptide positions 4, 6, and 9. Each of these anchor residues within the peptide interacts with a combination of amino acids present in the HLA II-binding groove (Dai et al. 2010). These polymorphic HLA amino acids, able to coordinate the peptide anchor residues, can be grouped into several binding pockets (Sturniolo et al. 1999). Different HLA binding grooves are formed by a linear combination of binding pocket variants. Understanding the relations between different HLA II proteins in terms of their binding affinities still presents a considerable challenge, since the high level of polymorphism of HLA II molecules makes the problem difficult to solve.

However, few attempts were made for HLA supertype classification (Sette and Sidney 1999; Castelli et al. 2002; Greenbaum et al. 2011) and HLA class II prediction (Karpenko et al. 2005; Doytchinova and Flower 2003). In most cases, the methods were trained and evaluated on very limited datasets, including only a single or a few different HLA class II alleles, and used either binding assay data (Sette and Sidney 1999; Castelli et al. 2002; Greenbaum et al. 2011), or the sequence or structure similarity of HLAs proteins (Lund et al. 2004; Doytchinova and Flower 2005). This motivated us to make a contribution to the problem by providing a stable group of supertypes and a predictor for HLA II-binding peptides, after the analysis of 27 HLA II proteins. Therefore, in this paper, the supertype classification was performed on the binding and motif-related information datasets of 27 HLA II proteins in order to find the results of consensus classification between them. This was done using *p* values based on multiscale bootstrap resampling hierarchical clustering (Shimodaira 2002, 2005). To confirm the biological relevance of earlier clustering results, the phylogenetic tree was computed. The overlap of the binding events was found to show large promiscuity in the HLA II–peptide interactions. Moreover, a very low rate of locus-specific binding events was observed for the HLA-DP locus. Finally, a well-known supervised classifier, namely a support vector machine (SVM) (Vapnik 1995), was trained to design the predictor for recognizing HLA II-binding peptides using only their sequence composition. The performance of the predictor was demonstrated by comparison with random forest (Breiman 2001), naïve Bayes (George and Langley 1995), artificial neural network (MacKay 1992; Bishop 1996), and K-nearest neighbor (Cover and Hart 1967) classifiers in terms of average precision, recall, specificity, accuracy, F-measure, and area under the ROC curve (AUC) values of random subsampled dataset. In addition, superiority of the predictor was also validated by leave-one-out cross-validation (LOOCV).

## Materials and methods

**Fig. 1:**
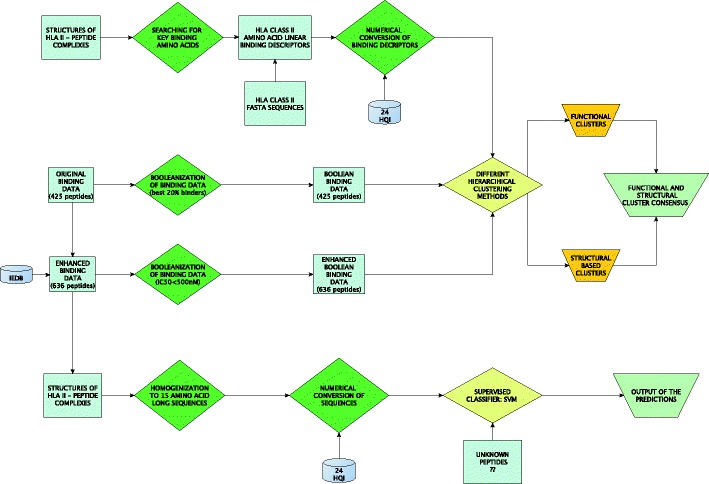
A block diagram of the workflow

The HLA supertype classification groups different HLA proteins into distinct classes on the basis of the given similarity parameters. The classification was performed considering both functional binding and structure-related information. The consensus between those two different approaches was proposed in order to identify some smaller groups of proteins that were correlated both functionally and structurally. Our workflow is presented on Fig. 1.

### Experimental binding affinities

Two different HLA binding datasets were used:

1. Greenbaum dataset consisting of 27 HLA II proteins binding 425 peptides obtained from *Phleumpratense* (Greenbaum et al. 2011)
2. An enhanced dataset containing the previously known protein–peptide pairs with additional binding data of 211 peptides for the same HLA repertoire (Immune Epitope Database^1^).

Both datasets contained the IC50 binding values of the binding affinity between HLAs and peptides. The raw datasets were transformed into binary binding matrices containing the value *1* for binding events and *0* for non-binding events. For the original Greenbaum dataset, the Greenbaum’s threshold criteria were maintained by considering the smallest 20 % of IC50 binding values as binding events for each HLA II. In this case, we could see that the maximum IC50 values were around 500 nM for considering a peptide as a binder to the HLA II protein. Hence, for the enhanced dataset, a compatible criterion was adopted by setting the threshold value at 500 nM. However, the usual IC50 binding threshold values for HLA I proteins were measured around 500 nM (Greenbaum et al. 2011) and the binding threshold for HLA II was generally 1,000 nM. These stringent IC50 threshold values were adopted in order to decrease the background noise of the data.

### Design of binding site composition

In this paper, inedited, simple, reliable, and informative structure-based linear motifs of the HLA II-binding sites are proposed. The aim was to obtain motif-based clusters to be compared with the clusters coming from the measured binding affinity data. Seven different HLA class II PDB structures in complex with peptides (1BX2, 1D5M, 1D5X, 1D6E, 3LQZ, 1UVQ, and 1JK8) were analyzed to investigate which amino acids within a protein sequence were responsible for binding. The HLA II residues were considered to interact with the peptides when the distance between any atom in the peptide residue and any atom in the HLA residue is less than or equal to a cutoff distance of 4 Å according to Mohanapriya et al. (2009). Fifteen different conserved residue positions, distributed over four different binding pockets of the HLA β-chains, were found to play a major role in the HLA II–peptide interaction in accordance with earlier literature (Doytchinova and Flower 2005; Patronov et al. 2011). The amino acids identified as principal “binding actors” were merged into linear binding motifs of 15 amino acid (AAs) long motifs containing all the amino acids essential for the binding. This is the simplest way to encode a complex physicochemical pattern of the active site into the usable linear motifs. Since the majority of the polymorphisms are located in the HLA β-chain, only the key positions within HLA II β-chains were considered. The following positions were used to generate binding motifs: β9, β11, β13, β28, β30, β37, β47, β57, β60, β61, β67, β70, β71, β74, and β78.

The binding motifs were then converted into numerical descriptors by representing the physicochemical features of each amino acid. For this purpose, we used the recently proposed set of 24 high-quality amino acid indices (HQI24) (Saha et al. 2011; Plewczynski et al. 2012). These numerical vectors were then used in the clustering analysis.

### Phase 1: clustering of functional and motif data

**Fig. 2:**
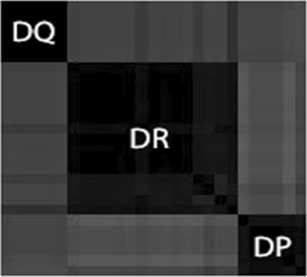
The true cluster plot of 27 HLA II proteins after performing *p* value-based multiscale bootstrap resampling hierarchical clustering. Three different clusters are clearly visible, corresponding to the three different loci DQ, DR, and DP

**Fig. 3:**
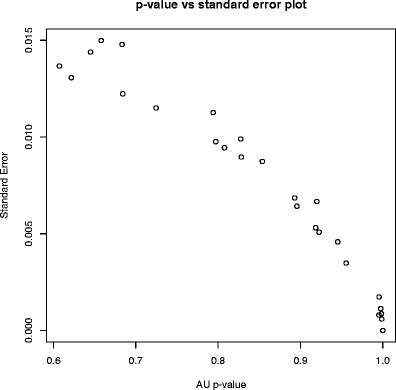
The plot of *p* values versus standard error for the binding data (636p) with the threshold IC50 < 500 nM. The standard error of the multi-bootstrap AC value is lower than 0.015 for the large majority of the cases, assuring a good cluster reliability. Similar results were found in the other datasets

The *p* value-based multiscale bootstrap resampling hierarchical clustering reveals the presence of three major clusters corresponding to the three HLA loci (DR, DQ, and DP) for both functional and motif datasets. Figure 2 shows that by the true cluster plot. The clusters show the expected difference between HLAs belonging to different loci in terms of peptide-binding affinity, but they do not give any information about possible clusters present within the same genetic locus. In order to investigate a possible functional intra-locus clustering, *p* value-based multiscale bootstrap resampling hierarchical clustering was performed with the above configuration. As shown in Fig. 3, almost all clusters have standard errors smaller than 0.015, assuring the high confidence level (99.985 %) of the approximately unbiased (AU) *p* values.

**Fig. 4:**
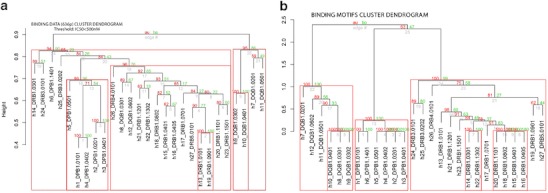
A dendrogram of 27 HLA II proteins for **a** 636 peptide binding data of threshold < 500 nM and **b** structure-based binding motifs, after performing *p* value-based multiscale bootstrap resampling hierarchical clustering

In *p* value-based multiscale bootstrap resampling hierarchical clustering, the *pvclust* function (Suzuki and Shimodaira 2006) was applied using R package^2^. Different clusters were generated by the *pvrec* function, which is marked by the red box in Fig. 4, with a gradual decrease of the AU-related cutoff value. With the use of these clustering functions, the functional binding and motif datasets of 27 HLA II proteins were clustered. For functional binding data, the Jaccard (1901) binary distance function was used, whereas the Euclidean distance was used for motif dataset. Moreover, *p* value-based multiscale bootstrap resampling hierarchical clustering uses the multi-level bootstrap analysis with a confidence value *α* iteratively lowered by a factor of 0.1 for each iteration. The goal of this action was to operate the clustering starting from highly reliable clusters with a gradual reduction of the similarity restrains between HLAs. The selection of the distance function was made based on the nature of the datasets. Figure 1 provides the block diagram of the resulting clustering procedure in phase 1.

### Phase 2: prediction of HLA II-binding peptides

A support vector machine (Vapnik 1995) classifier was used to predict whether or not a peptide binds to an HLA type II protein. The entire pool of 636 peptides was initially transformed into the numerical domain using the HQI24 representation of residues, i.e., 24 high-quality amino acid indices (Saha et al. 2011; Plewczynski et al. 2012). The length of all the peptides was homogenized to 15 AA, cutting the less relevant bordering amino acids of a few 16mer and 17mer peptides present in the dataset. The dissection was selected after an accurate comparative analysis of the less conserved residues within longer peptides. A multiple sequence alignment was performed among proteins containing the peptides to be homogenized in length. The amino acids present in the positions less conserved within these alignments were removed. The homogenization of the peptides length was a mandatory step for SVM.

The binary binding affinity matrix was used to define the total number of binding events for each given peptide. The percentage of positive activity (PPA) was defined for this purpose. The highest number of positive activity was computed and considered as a reference value of 100 %. The rest of the peptides’ PPA were then computed with respect to the highest PPA. A threshold was defined a priori. If the PPA is greater than the pre-defined threshold, then the activity for that peptide is equal to 1, otherwise it is equal to 0. Each activity value indicates whether or not the peptide is an “HLA binder.” Since the activity value of a peptide is defined with respect to the threshold value, hence a lower threshold gives a higher number of binding peptides. Different threshold values were applied and the statistics is given in Table 1. Moreover, it was observed that the number of positive and negative binders plays a crucial role for supervised classifiers. Hence, the threshold level at 30 % was considered for balanced number of binders.

**Table 1:** Statistics of the dataset used for different classifiers is marked in bold

Threshold levels (%)	Number of positives	Number of negatives	Percentage of positives
10	535	101	84
15	459	177	72
20	415	221	65
**30**	**310**	**326**	**49**
40	244	392	38
50	156	480	25

In this paper, we implemented random subsampling validation, to estimate the unbiased error rate of the designed technique. This method randomly splits the dataset into training and test (validation) data. For each of such split, the classifier learned the training data, and predictive accuracy was assessed using the test data. The results were then averaged over multiple such splits. For the 30 % threshold level, the training and test samples for positive instances were populated in the ratio of 4:1 from all available positive samples. The number of negative samples for each type was similarly chosen. Hence, two thirds of the dataset was used for training and one third for testing. Random subsampling produces better error estimates than a single train-and-test split. The advantage of this method over *k*-fold cross-validation is that the proportion of the training/validation split is not dependent on the number of iterations or folds. In this work, we performed three random splits in the positive/negative datasets for 30 % threshold level. This was done to eliminate the possible bias during the training procedure in any given train/test dataset combination. Using a support vector machine classifier, this threshold was trained separately on the three randomly chosen independent test datasets and was then tested to compute precision, recall, specificity, accuracy, F-measure, and AUC values. The phase 2 classification task is illustrated in Fig. 1.

## Results and discussion

Different methods and datasets show comparable results with some expected differences (Greenbaum et al. 2011). The variability of the clusters seems to be more influenced by the dataset rather than by the methods or the threshold values used. The final functional data classification was chosen considering the clustering results and analyzing the consensus between them, by visual inspection.

### Functional supertype classification

Figure 4a shows eight different functional supertypes that were identified. For the HLA-DP proteins, a single functional supertype containing five strongly correlated HLA proteins (DPB1*0101, DPB1*0201, DPB1*0402, and DPB1*0501) was described in all the cases. The DPB1*1401 is the only protein to be clustered in a supertype belonging to another genetic locus (HLA-DR), in accordance with Greenbaum et al. We observed a single supertype for HLA-DP proteins. The lack of any refined cluster structure within the HLA-DP locus is in opposition to Greenbaum’s functional classification of the HLA-DP proteins (Greenbaum et al. 2011), where two different supertypes were proposed.

For the HLA proteins, belonging to the DQ locus, three major supertypes were found, each containing two proteins: (DQB1*0302, DQB1*0401), (DQB1*0201, DQB1*0501), and (DQB1*0301, DQB1*0602). Different possible classifications can be made for the DR locus, according to the variety of AU values of the dendrograms. In this case, three constant clusters were recognized (DRB1*0401, DRB1*0405, DRB1*0802), (DRB1*1302, DRB3*0101, DRB3*0202), and (DRB1*0101, DRB1*0901). The other proteins are not stable within the same clusters under different conditions.

### Motif-based supertype classification

**Fig. 5:**
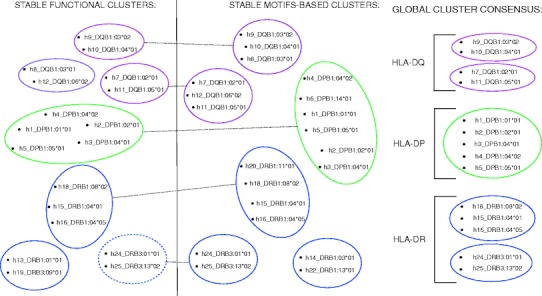
**a** Stable clusters found by *p* value-based multiscale bootstrap resampling hierarchical clustering from binding affinity and motif datasets. **b** Consensus results of those clusters

A detailed analysis of the clustering results leads us to the identification of the seven different motif-based supertypes as shown in Fig. 4b. All the proteins belonging to the DP genetic locus (DPB1*0101, DPB1*0201, DPB1*0401, DPB1*0402, DPB1*0501, and DPB1*1401) were grouped into a single supertype, similarly to the functional case, examined in the previous section. DQ proteins were grouped into two different supertypes, each containing three HLAs: (DQB1*0301, DQB1*0302, DQB1*0401) and (DQB1*0201, DQB1*0501, DQB1*0602). As in the functional case, the motif-based classification of the DR proteins is less defined with respect to the other loci. The HLA-DR can be grouped into four supertypes: (DRB1*0401, DRB1*0405, DRB1*0802, DRB1*1101), (DRB3*0101, DRB3*0202), (DRB1*0301, DRB1*1302), and the fourth containing the remaining proteins. Visualization of clusters is shown in Fig. 5a. In both functional and motif-based clustering, multiple calculation methods were applied. Partially overlapping results are shown in Fig. 5a. The clustering overlaps found between these two datasets are defined as stable clusters. Thereafter, only these stable clusters were used in the final consensus between the functional and motif-based supertype classifications.

### Consensus between supertype classifications

In both clusterings, all the HLAs were classified within the same loci with the exception of the DPB1*1401 HLA-DP protein which belong to the DR type just for the functional classification. A more relevant partial overlapping of the stable intra-locus clusters was found via consensus selection between common binding and motif-based clusters. For HLA-DP proteins, five (of six) were found to maintain a very strong correlation in both cases by belonging to the same cluster in opposition to the Greenbaum observation. These proteins are: DPB1*0101, DPB1*0201, DPB1*0401, DPB1*0402, and DPB1*0501. The consensus between proteins of the DQ locus reveals two common groups, one of them containing DQB1*0302 and DQB1*0401 and the other containing two HLAs, DQB1*0201 and DQB1*0501. Figure 5b shows the details of the consensus results.

### Results of phylogenetic tree analysis

**Fig. 6:**
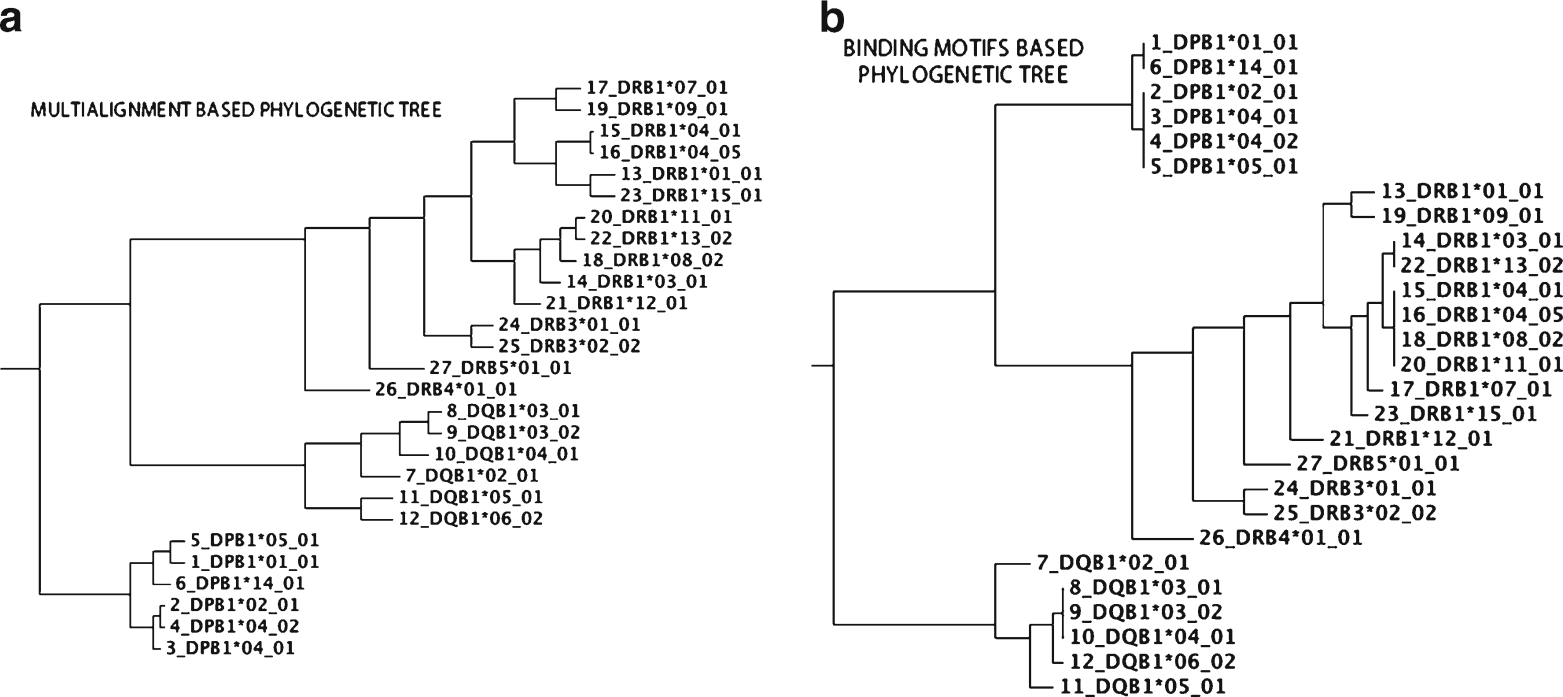
Phylogenetic trees generated from the multi-alignment of **a** entire HLA sequences and **b** structure-based binding motifs

Phylogenetic multi-alignment-based trees were created for both binding motifs and HLAs multi-fasta files. The Clustalw 2.1 (Larkin et al. 2007) software was used for this purpose and the unweighted pair group method with arithmetic mean clustering algorithm was chosen to build the phylogenetic trees. Both phylogenetic trees divide the HLAs into three different loci: DR, DQ, and DP. Moreover, the proteins grouped via functional/motifs consensus were also similarly correlated in the phylogenetic trees. The tree generated from the binding motifs data shows a better correlation with the HLA–peptide binding data classification and the motifs/binding consensus groups collected. This suggests that the motif description used here is able to collect good structural/sequence information in the vicinity of the binding site, thus lowering the background noise present in the global HLA protein sequences. Phylogenetic trees are shown in Fig. 6.

### Peptide-binding specificity

**Fig. 7:**
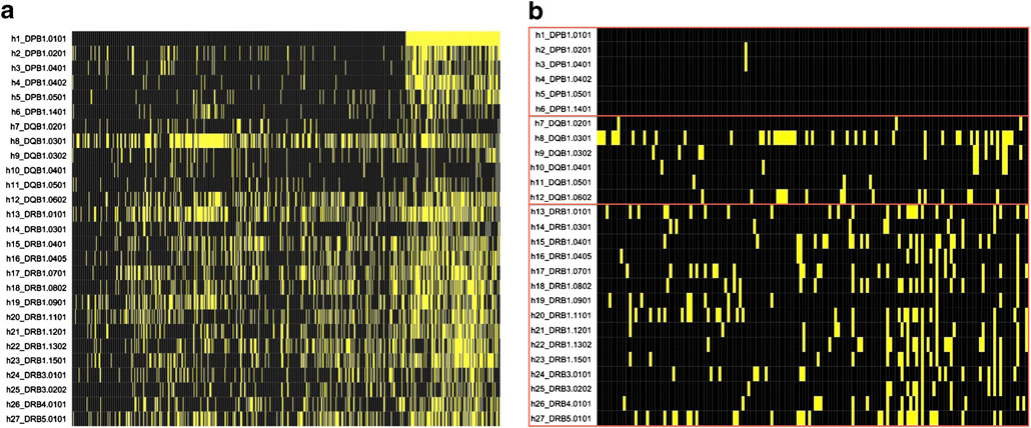
The heat map of the HLA–peptide binding event where each *yellow line* represents a binding event. While in **a** all the peptides were considered for binding, in **b**, only 1 of the 149 peptides, binding HLAs and belonging to the same locus, was considered. Note the lack of binding related to the HLA-DP family

A binary heat map was drawn to visualize the peptide preferential specificity to the different HLAs Fig. 7a. It shows a general high peptide overlapping, suggesting high flexibility in the HLA II peptide-binding events as described in literature (Yaneva et al. 2009). The number of peptides that binds HLAs and belongs to more than one locus was calculated. The percentage of those peptides for the 636 pep Boolean table with an IC50 cutoff value of 500 nM is equal to 76.58 %. Only a small part of the peptides (23.42 %) bind exclusively HLAs within the same locus. The binding data concerning these “locus-specific” peptides were plotted into a second binary heat map shown in Fig. 7b, which shows only the binding events occurring within the same loci. Interestingly, locus-specific binding events concerning the HLA-DP are almost lacking. This observation is statistically relevant since the main percentage of binding events, with respect to the maximum theoretical binding are, respectively, 3.8 % for DR, 2.17 % for DQ, and merely 0.05 % for DP. This lack of HLA-DP-specific binding events, together with the wide functional/motif consensus found among the HLA-DP proteins, conveys the possible presence of one (or more) highly conserved binding groove with low peptide specificity in the DP protein family. Further docking analyses are required to test this hypothesis. The HLA–peptide binding frequencies (see Fig. 7) show the number of binding events collected by each peptide for different HLA loci (DR, DQ, and DP).

### Performance of SVM-based HLA II-binding peptide predictor

The performance of a SVM classifier based predictor is described here using precision (*P*), recall (*R*), specificity (SP), accuracy (*A*), F-measure (F1), and AUC values. Please note that the computational procedure of these metrics is mentioned in the Electronic supplementary material. In this predictor, the radial basis function kernel is used for SVM. Here, the parameters of the kernel function, *γ*, and the trade-off between the training error and the margin C, are set to be 0.5 and 2.0, respectively.

The problem of overfitting is addressed by training SVM on independent test datasets. Three random runs of the training and test sample sets were considered to generate precision, recall, specificity, accuracy, and F-measure values for designing the software tool. Average test set accuracies are reported in Table 2. The LOOCV often works well to estimate the generalization error for continuous error functions such as the mean squared error, but it is usually very expensive from the computational point of view because the training process must be repeated many times. However, here size of the training datasets suits LOOCV methodology. Hence, LOOCV has performed to establish the superiority of the SVM predictor.

**Table 2:** Performance comparison of SVM-based HLA II–peptide predictor with other supervised classifiers at 30 % threshold level in terms of average precision, recall, specificity, accuracy, F-measure, and AUC

Algorithms	Precision (*P*)	Recall (*R*)	Specificity (SP)	Accuracy (*A*)	F-measure (*F*_1_)	AUC
SVM	60.07	88.33	87.62	76.26	71.48	0.75
RF	58.63	85.02	84.32	74.14	69.22	0.74
NB	57.04	83.85	83.87	73.64	67.85	0.73
ANN	55.32	82.07	81.74	71.73	65.32	0.71
K-NN	51.86	80.72	79.98	69.85	63.04	0.68

*RF* random forest, *NB* Naïve Bayes, *ANN* artificial neural network, *K-NN* K-nearest neighbor

The performance analysis shows that SVM results in terms of precision, recall, specificity, and accuracy and F-measure values are significantly better in percentage of finding true positive and true negative at 30 % threshold level. Moreover, the results of other classifiers are low in comparison with SVM, as reported in Table 1. Here, it was observed that lower thresholds create the overfitting problem by producing similar precision and recall values. Therefore, at 30 % threshold level, the SVM-based predictor predicting a peptide as a binder, while requiring a smaller number of binding events, results in a better HLA binding classification. The results in Table 1 show a good prevision capability of the predictor, suggesting that its optimization could provide a potentially valuable instrument for discovering HLA class II binding epitopes, the issue that is of great importance in vaccinology. Optimization strategies planned for this method include the consensus of multiple SVMs each trained both on the general locus type and on the functional/motif consensus groups, via the clustering analysis as described above.

## Conclusions

Functional and motif-based clustering of 27 defined HLA class II complexes were performed by revealing the presence of proteins sharing both functional and structural properties, supporting the concept of supertype. New binding motifs based on structural information were proposed for this purpose. We address it as a potentially good instrument for the description of interactions in a typical bioinformatical analysis. During the clustering analysis, a large overlap of HLA-specific binding events was found which confirms the high binding promiscuity present in the HLA class II proteins. Surprisingly, a general lack of locus-specific binding events was observed in the HLA-DP proteins. A high motif-based/functional correlation between these proteins was found as well, suggesting the possible presence of common and low specific binding patterns between them. Preliminary docking studies confirmed this theory, which to the best of our knowledge had not been reported before. Finally, an SVM-based HLA II–peptide binding predictor was developed. The results show that this predictor is a potentially good candidate for vaccinology studies.

## Electronic supplementary material

Below is the link to the electronic supplementary material.

ESM 1 (DOCX 145 kb)

ESM 2 (XLS 22 kb)

ESM 3 (XLS 169 kb)

ESM 4 (TXT 0 kb)

ESM 5 (TXT 84 kb)

ESM 6 (TXT 139 kb)

ESM 7 (TXT 27 kb)

ESM 8 (TXT 84 kb)

ESM 9 (TXT 139 kb)

ESM 10 (TXT 27 kb)

ESM 11 (TXT 43 kb)

ESM 12 (TXT 75 kb)

ESM 13 (TXT 15 kb)

ESM 14 (TXT 1,037 kb)

## Footnotes

1
www.immuneepitope.org/

2
http://www.r-project.org
