# Supplementary material for: Consensus classification of human leukocyte antigen class II proteins
Source: Immunogenetics. 2012 Nov 16;65(2):97–105. doi: 10.1007/s00251-012-0665-6 (PMC3543608; doi:10.1007/s00251-012-0665-6)
Supplement: Supplementary file 1 — (DOCX 145 kb) [file 251_2012_665_MOESM1_ESM.docx]

Consensus classification of Human Leukocyte Antigens class II protein

**Indrajit Saha, Giovanni Mazzocco, and Dariusz Plewczynski**

Supplementary Materials

**Results of Average Linkage Hierarchical Clustering**


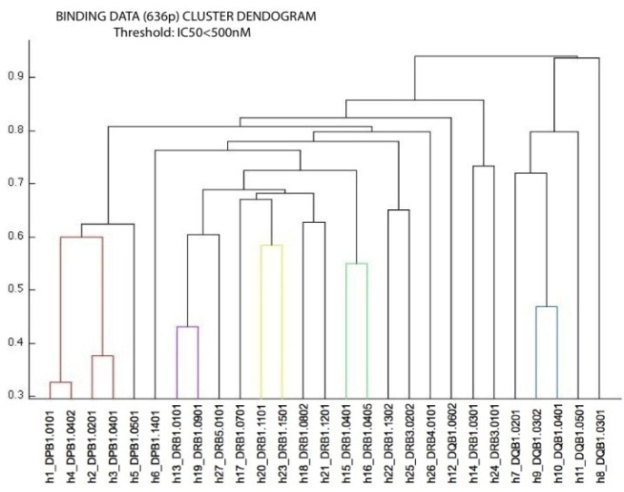
The function *colorthreshold* was applied to the average linkage hierarchical clustering, changing the threshold value *t* iteratively. This function assigns a unique color to each group of nodes in the dendrogram where the linkage value is less than a threshold *t*, defined by the operator. The colored clusters were generated using the thresholds values *t=0.65* and t=*0.6*, respectively.

(a)


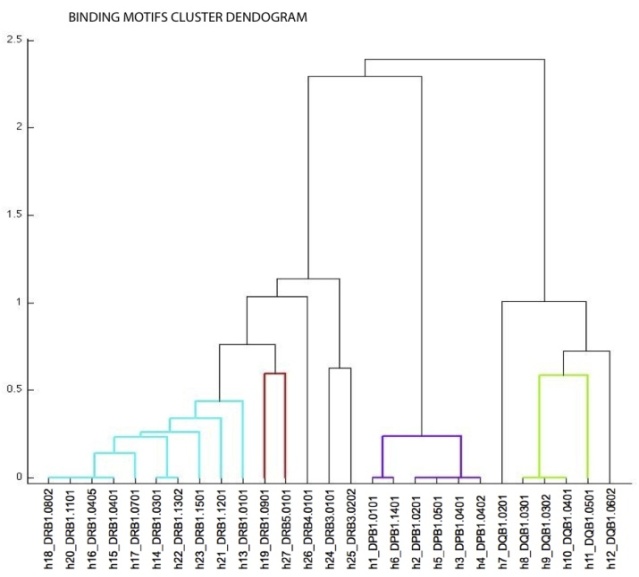


(b)

**Fig.** Dendrogram 27 HLA II proteins for (a) 636 peptide binding data of threshold < 500nM , (b) structural-based binding motifs, after performing average linkage hierarchical clustering.

**Performance Measure of SVM based Predictor**

The performance of the support vector machine (SVM) classifier based predictor is described here using precision (*P*), recall (*R*), specificity (*SP*), accuracy (*A*) and (*F_1_*) values. The error estimates are calculated using the leave-one-out procedure using the following equations.

(1)

$$P=\frac{TP}{TP+FN}\times100\%$$

(2)

$$R=\frac{TP}{TP+FN}\times100\%$$

(3)

$$SP=\frac{TN}{FP+TN}\times100\%$$

(4)

$$A=\frac{TP+TN}{TP+FP+FN+TN}\times100\%$$

(5)

$$F_{1}=\frac{2TP}{2TP+FP+FN}\times100\%$$

where TP is the number of true positives, FP is the number of false positives, TN is the number of true negatives and FN is the number of false negatives. The classiﬁcation accuracy A provides an overall accuracy measure, whereas recall R measures the percentage of correct predictions (the probability of correct prediction), precision P gives the percentage of observed positives that are correctly predicted (the measure of the reliability of positive instances prediction) and specificity SP gives the percentage of true negative rate (TNR). F1 score (also F-score or F-measure) is a measure of a test’s accuracy. F1 score reaches its best value at 1 and worst score at 0.
